# Diagnosis by real-time polymerase chain reaction of pathogens and antimicrobial resistance genes in bone marrow transplant patients with bloodstream infections

**DOI:** 10.1186/1471-2334-13-166

**Published:** 2013-04-05

**Authors:** Liana Carballo Menezes, Talita Trevizani Rocchetti, Karen de Castro Bauab, Paola Cappellano, Milene Gonçalves Quiles, Fabianne Carlesse, Jose Salvador Rodrigues de Oliveira, Antonio Carlos Campos Pignatari

**Affiliations:** 1 Special Clinical Microbiology Laboratory (LEMC), Federal University of São Paulo/UNIFESP, São Paulo, Brazil; 2 Division of Infectious Diseases, Federal University of São Paulo/UNIFESP, São Paulo, Brazil; 3 GRAACC – Grupo de Apoio ao Adolescente e à Criança com Câncer (Support Group for Children and Adolescents with Cancer), São Paulo, Brazil; 4 Division of Hematology, Federal University of São Paulo/UNIFESP, São Paulo, Brazil

**Keywords:** Bone marrow transplants, Real-time polymerase chain reaction, Antibiotic resistance genes, Bloodstream infections

## Abstract

**Background:**

Early identification of pathogens and antimicrobial resistance in bloodstream infections (BSIs) decreases morbidity and mortality, particularly in immunocompromised patients. The aim of the present study was to compare real-time polymerase chain reaction (PCR) with commercial kits for detection of 17 pathogens from blood culture (BC) and 10 antimicrobial resistance genes.

**Methods:**

A total of 160 BCs were taken from bone marrow transplant patients and screened with Gram-specific probes by multiplex real-time PCR and 17 genus-specific sequences using *TaqMan* probes and *bla*SHV, *bla*TEM, *bla*CTX, *bla*KPC, *bla*IMP, *bla*SPM, *bla*VIM, *van*A, *van*B, and *mec*A genes by SYBR Green.

**Results:**

Twenty-three of 33 samples identified by phenotypic testing were concordantly positive by BC and real-time PCR. Pathogen identification was discordant in 13 cases. In 12 of 15 coagulase-negative staphylococci, the *mecA* gene was detected and four *Enterococcus* spp. were positive for *vanA*. Two *bla*CTX and three *bla*SHV genes were found by quantitative PCR. The *bla*KPC and metallo-β-lactamase genes were not detected. Five fungal species were identified only by real-time PCR.

**Conclusions:**

Real-time PCR could be a valuable complementary tool in the management of BSI in bone marrow transplants patients, allowing identification of pathogens and antimicrobial resistance genes.

## Background

Approximately 60% of episodes of fever in patients with neutropenia are frequently correlated with documented bloodstream infection (BSI). BSI is associated with high morbidity and a mortality rate ranging from 20 to 70%. The SENTRY Antimicrobial Surveillance Program (1997–2002) reported that 10 bacterial species accounted for 89–92% of all isolates in 659,935 cases of sepsis reported in the United States in 2000. The ranking of microorganisms was similar across North America, Latin America, and Europe [1]. The rank of the five major pathogens in the Brazilian SCOPE (Surveillance and Control of Pathogens of Epidemiological Importance) were *Staphylococcus aureus* (15.4%), coagulase-negative staphylococci (CoNS) (13.4%), *Klebsiella* spp. (13.2%), *Acinetobacter* spp. (12.5%), and *Pseudomonas aeruginosa* (8.9%) [2].

The most dangerous clinical manifestations of BSI are sepsis and shock, which are the 10th leading cause of death in the United States, accounting for 6% of all deaths (50.37 deaths per 100,000 individuals in the overall population) [3]. Early diagnosis of sepsis and provision of appropriate antimicrobial therapy correlate with positive clinical outcomes [4]. Blood culture (BC) is considered to be the gold standard for detecting microorganisms in the bloodstream, including those that have antibiotic resistance genes [5]*.* Molecular amplification techniques have been used to detect microorganisms in BSI. Automated BC systems take 1–2 days, on average, to signal a positive result and a further 1–2 days to finalize bacterial identification and antimicrobial testing. Rapid detection of bacterial resistance mechanisms in BC bottles can assist physicians with both patient management and infection control.

## Methods

### Patients

During October 2008 to July 2011, 160 BCs from Bactec bottles were analyzed from 33 immunocompromised patients (31 adults and two children) with hematological malignancies who underwent bone marrow transplantation (seven acute myeloid leukemia, six multiple myeloma, six non-Hodgkin’s lymphoma, four acute lymphoblastic leukemia, two Hodgkin’s disease, two chronic myeloproliferative disorders, one chronic lymphoblastic leukemia, one adenovirus dystrophia, one Ewing syndrome, one pineal carcinoma and one testicular cancer). Patients were admitted to the Institute of Pediatric Oncology (GRAACC) and the Adult Hematology Unit of São Paulo Hospital, Federal University of São Paulo, Brazil. Informed consent was obtained from patients and the study was approved by the Clinical Research Ethics Committee of the hospital.

### Sample collection and bacterial identification

The samples were collected as part of standard patient care from patients suspected of BSI in the pretransplant mobilization phase of hematopoietic stem cell transplantation, transplanted patients, and patients admitted with clinical complications after transplantation. At the onset of fever (>37°C) or in the presence of any clinical symptom compatible with infection, two sets of BCs were taken. BSI was defined as the isolation of a bacterial or fungal pathogen from at least one BC. For CoNS, corynebacteria other than *Corynebacterium jeikeium* and common skin contaminants, at least two sets of positive BCs were required. All episodes of BSI were then subclassified as single-agent (Gram-positive, Gram-negative or fungal) or polymicrobial. For polymicrobial BSI, two or more pathogens were isolated from a single BC or at least two separate BCs at 96 h apart. BCs were performed using the Bactec 9240 (Becton Dickinson, Microbiology Systems, Cockeysville, MD, USA). BC bottles were signaled positive by Bactec 9240 after detection of bacterial growth. The samples were negative by Bactec when no bacterial growth was detected after 5 days incubation. Identification to species level and susceptibility testing were performed using the automated system Phoenix 100 (Becton Dickinson Microbiology Systems).

### DNA extraction

Total DNA was extracted from the positive or negative 500-μl BC broth after incubation on Bactec 9240, using the phenol–chloroform method (Brazol; LGC Biotechnologia, Cotia, Brazil) with 300 mg glass beads (0.3 mm diameter; Scientific Industries, Bohemia, NY, USA) and processed in a disruptor Genie (Scientific Industries) for 10 min to achieve cell lysis.

### Primers and *TaqMan* probe

The primers used for detection of the resistance genes, *bla*SHV, *bla*TEM, *bla*CTX-M, *bla*IMP, *bla*SPM, *bla*VIM, *bla*KPC, *van*A, *van*B and *mec*A, have been described previously [6]. The *TaqMan* probes for multiplex real-time polymerase chain reaction (PCR) detection of Gram-positive and Gram-negative bacteria have been previously described, as well as species-specific *TaqMan* probes for detection of *Enterococcus* spp., CoNS, *S. aureus*, *Streptococcus pneumoniae*, *Escherichia coli*, *Klebsiella pneumoniae*, *Enterobacter cloacae*, *Proteus mirabilis*, *Salmonella* spp., *Serratia marcescens*, *Acinetobacter baumannii*, *P. aeruginosa*, *Stenotrophomonas maltophilia*, *Mycobacterium tuberculosis*, *Aspergillus* spp., *Candida* spp. and *Fusarium* spp. [7]. All the primers and probes were selected from the National Center for Biotechnology Information website (NCBI; http://www.ncbi.nlm.nih.gov) and synthesized by Applied Biosystems (Foster City, CA, USA). The primers were checked for specificity in a BLAST search available through the NCBI website (http://blast.ncbi.nlm.nih.gov/Blast.cgi). A primer set for the hemochromatosis gene was designed to be used as an internal control.

### Real-time PCR

Detection of bacterial DNA was screened using universal primers of 16S rDNA gene. Differentiation between Gram-positive and Gram-negative bacteria was done by *TaqMan* multiplex real-time PCR [7]. Differentiation of the other genes was performed by monoplex real-time PCR. Amplification for *TaqMan* probes reactions was performed in a 20-μl reaction volume, using 10 μl *TaqMan* Universal Master Mix 2X (Applied Biosystems), 5 μl template DNA, 0.5 μM each primer, and 0.3 μM probe. The real-time PCR conditions were as follows: 50°C for 2 min and 95°C for 10 min; 40 cycles of 95°C for 15 s and 60°C for 60 s. Resistance genes amplification by *SYBR Green* monoplex real-time PCR was performed in a 25-μl reaction mixture containing 12.5 μl Platinum SYBR Green qPCR SuperMix (Invitrogen, Carlsbad, CA, USA), 0.5 μM each primer, and 5 μl purified DNA extracted from samples. The real-time PCR conditions were as follows: 50°C for 2 min and 95°C for 10 min; 40 cycles of 95°C for 15 s and 60°C for 60 s; and a melting curve step (from 68°C to 95°C in increments of 0.5°C/s). The metallo-β-lactamase amplification was performed by multiplex real-time PCR [7]. The ABI 7500 real-time PCR System (Applied Biosystems) instrument was quantified online and at the endpoint with the sequence detection system software (version 2.0; Applied Biosystems).

### Data analysis

Data collection and statistical analysis were performed using SSPS version 17.0 software (SPSS Inc., Chicago, IL, USA) and Microsoft Office Excel 2007 (Microsoft, Redmond, WA, USA). The area under the receiver operating characteristic curve (AUC) was determined for binary diagnostic test results for all genes. Comparison of the BC identification by Phoenix versus real-time PCR results were evaluated by *χ*^2^ tests. Discordance between the results from the two methods was assessed using McNemar’s test (statistical significance was set at a two-tailed exact test, based on the binomial distribution with p = q = 0.5). The κ statistic was calculated to measure the level of agreement between BC by Phoenix and real-time PCR results.

## Results

### Sample collection and bacterial identification

A total of 160 blood samples collected from 33 patients were evaluated for BSI. Thirty-three samples, representing 15 febrile episodes, tested positive by BC. BC identified 23 Gram-positive bacteria (15 CoNS, four *Enterococcus faecium*, two *Enterococcus faecalis* and two *Str. pneumoniae*) and 10 Gram-negative bacteria [one *Pseudomonas putida,* two *Acinetobacter* spp., three *Ent. cloacae*, one *K. pneumoniae*, one *E. coli*, one non-fermenting Gram-negative bacilli (NFGNB) and one *Citrobacter freundii*].

### Real-time PCR

Real-time PCR detected 37 positive samples (Table 1). The data for negative samples by the two methods are not shown. Twenty-three of 33 samples identified by phenotypic testing were concordantly positive by BC and real-time PCR (Table 1). Twenty-one samples were discordant by both methods. Nine BC-positive samples were negative by real-time PCR and 12 BC-negative samples were positive by PCR.

**Table 1:** Results of real-time PCR, culture identification and antimicrobial susceptibility of positive samples from BC bottles

**Patients**	**Date samples**	**Bactec® Results**	**Bacterial isolated from blood culture (Phoenix®)**	**Bacterial detected with the PCR test**	**Antimicrobial susceptibility (Phoenix®)**		**Resistance genes by real-time PCR**
	10/9/2009	Positive	*S. epidermidis*	CoNS	S*	Oxacilin	*mec*A
	10/9/2009	Positive	*S. epidermidis*	CoNS	S	*Oxacilin*	*mec*A
	10/9/2009	Positive	*S. epidermidis*	CoNS	S	*Oxacilin*	*mec*A
						Cephalosporins	
01	12/20/2009	Positive	*C.freundii*	CoNS	S	and	ND
						Carbapenems	
	12/20/2009	Positive	*S. epidermidis*	CoNS	R*	Oxacilin	*mecA*
	3/1/2009	Negative	ND	CoNS and GN	NA	NA	*bla*SHV
	1/9/2010	Negative	ND	*Aspergillus s*pp.	NA	NA	NA
	12/4/2008	Positive	*E. cloacae*	*E. cloacae*	R	*Cephalosporins*	ND
02	12/4/2008	Positive	*E. cloacae*	*E. cloacae*	R	*Cephalosporins*	ND
	12/4/2008	Positive	*E. cloacae*	*E. cloacae*	R	*Cephalosporins*	*bla*SHV
	3/5/2009	Positive	CoNS	CoNS	R	Oxacilin	*mec*A
03	3/5/2009	Positive	CoNS	CoNS	R	Oxacilin	*mec*A
	3/5/2009	Positive	CoNS	CoNS	R	Oxacilin	ND
	5/8/2009	Positive	*P. putida*	ND	NA	NA	ND
04	5/8/2009	Positive	*S. epidermidis*	CoNS	R	Oxacilin	*mec*A
	5/8/2009	Positive	*S. epidermidis*	CoNS	R	Oxacilin	*mec*A
05	8/2/2009	Positive	*S. pneumoniae*	ND	S	Penicilin	ND
	8/2/2009	Positive	*S. pneumoniae*	*S. pneumoniae*	S	Penicilin	ND
	6/6/2009	Positive	*E. faecium*	*Enterococcus* spp.	R	Glycopeptide	*van*A
06	6/6/2009	Positive	*E. faecium*	ND	R	Glycopeptide	*van*A
	6/6/2009	Positive	*E. faecium*	ND	R	Glycopeptide	*van*A
07	1/18/2009	Positive	NFGNB	*A. baumannii*	R	Cephalosporins and Carbapenems	ND
						Cephalosporins	
	1/31/2009	Positive	*Acinetobacter* spp.	ND	R	and	ND
						Carbapenems	
	1/31/2009	Negative	ND	*A. baumannii*	NA	NA	ND
					R	Cephalosporins	
08	3/2/2009	Positive	*K. pneumoniae*	ND	and	and	ND
					S	Carbapenems	
09	9/28/2009	Positive	*E. faecalis*	ND	S	Glycopeptide	ND
	9/28/2009	Positive	*E. faecalis*	*Enterococcus* spp.	S	Glycopeptide	ND
10	12/12/2008	Positive	*E. faecium*	*Enterococcus* spp.	R	Glycopeptide	*vanA*
	12/9/2008	Negative	ND	*Enterococcus* spp.	NA	NA	ND
11	12/18/2008	Negative	ND	*K. pneumoniae*	NA	NA	*bla*CTX-M
	12/21/2008	Negative	ND	*K. pneumoniae*	NA	NA	*blaCTX-M*
	2/21/2009	Negative	ND	*CoNS and GN*	NA	NA	*ND*
	28/03/2011	Negative	ND	*CoNS*	NA	NA	*ND*
	2/21/2009	Negative	ND	*Candida spp.*	NA	NA	*NA*
12	4/8/2011	Positive	S. epidermidis	*CoNS*	R	Oxacilin	*mecA*
	4/8/2011	Positive	S. epidermidis	*CoNS*	R	Oxacilin	*mecA*
	4/8/2011	Positive	S. epidermidis	*CoNS*	R	Oxacilin	*mecA*
13	3/14/2009	Negative	ND	*ND*	NA	NA	*blaSHV*
14	11/4/2010	Positive	A. baumannii	*A. baumannii*	S	Cephalosporins	*ND*
15	3/26/2011	Positive	E. coli	*E. coli*	S	Cephalosporins and Carbapenems	*ND*
16	3/31/2011	Positive	S. epidermidis	*CoNS*	R	Oxacilin	*mecA*
17	5/8/2009	Positive	CoNS	*ND*	R	Oxacilin	*ND*
	5/8/2009	Positive	CoNS	*ND*	R	Oxacilin	*ND*
18	5/14/2009	Negative	ND	*Aspergillus spp.*	NA	NA	*NA*
19	5/2/2009	Negative	ND	*Candida albicans*	NA	NA	*NA*
	5/4/2009	Negative	ND	*Candida albicans*	NA	NA	*NA*

*R*: Resistant; *S*: Susceptible; * CLSI 2010; *NA*: results not available; *ND*: Not Detected.

Table 1 shows only positive samples that were isolated by BC and/or real-time PCR. The other 21 negative results are not shown.

For Gram-positive bacteria, 15 CoNS were positive by BC and 13 of these were detected by real-time PCR. Three CoNS were detected by real-time PCR but did not grow in culture. Three of six BCs that were positive for *Enterococcus* spp. were negative by real-time PCR, while whereas BC did not detect one sample that was positive by real-time PCR. In two samples, BC identified *Str. Pneumoniae*, while real-time PCR was not positive for one of these samples.

For Gram-negative bacteria, all *Ent. cloacae* and *E. coli* samples were concordant by both methods (Table 1). In three samples, growth of *C. freundii*, *Acinetobacter* spp. and *P. putida* was detected by BC and by Gram-negative probe; however, specific primers and probes were not designed for these pathogens, therefore they were not considered discrepant. Two samples were positive for *K. pneumoniae* by real-time PCR and negative by BC, whereas one BC-positive sample was negative by real-time PCR. Three samples were identified as *A. baumannii* by real-time PCR and one of these was positive by BC, one was BC-positive for NFGNB, and one was not detected by BC.

The overall concordance level was 72.7% for phenotypic and real-time PCR methods with a Cohen κ coefficient of 0.7 (95% CI: 0.61–0.80).

For resistance genes, real-time PCR detected 12 CoNS with *mecA* genes*.* Nine samples were concordant for detection of the *mecA* gene compared with phenotypic resistance to methicillin. The *vanA* gene was detected in four *Enterococcus* spp. (*Enter. faecalis* and *Enter. faecium*) and none were positive for the *van*B gene. The concordance was 100% between two vancomycin resistance methods (Table 1).

For Gram-negative bacteria, five samples detected the ESβL gene by real-time PCR. The *bla*SHV gene was detected in three samples and *bla*CTX-M in two (Table 1). Only two of the five positive ESβL real-time PCR samples were identified by the phenotypic method as ESβL producers; the other two were identified as ESβL producers by the phenotypic method but were negative by real-time PCR (Table 1).

The *bla*KPC and metallo-β-lactamase genes were not detected. The *A. baumannii* samples showed carbapenem phenotypic resistance; however, the carbapenemase genes were not detected.

Three *Candida* spp. were detected from two patients by real-time PCR but not by BC. In one patient, two samples were positive by real-time PCR. Two samples were positive for *Aspergillus* spp. by real-time PCR with negative galactomannan antigenemia. *Fusarium* spp. were not detected.

Real-time PCR performance for bacterial identification was adjusted as follows: sensitivity, 78%; specificity, 93%; negative predictive value (NPV), 95%; positive predictive value (PPV), 72% when compared with the phenotypic method.

## Discussion

Early identification of the causative pathogen in BSI is crucial, especially in transplant patients, and can improve survival in the post-transplant period. In addition, rapid detection of resistance genes in the bloodstream can contribute to the efficacy of antimicrobial treatment with reduced morbidity/mortality [8-10].

The use of PCR for diagnostic purposes has established a new era in the detection and characterization of microorganisms directly from clinical samples. Several protocols based on PCR amplification of 16S rDNA for differentiation of Gram-positive and Gram-negative bacteria have been used with samples collected from different infectious sites [11,12].

Commercially available multiplex real-time PCR assay kits have been evaluated in adult patients with hematological malignancies and compared with BC for identification of microorganisms [13]. Our study reports the clinical application of real-time PCR for identification of bacteria and detection of antibiotic resistance genes in adult and pediatric patients with hematological malignancies.

The PCR showed nine negative results that tested positive by BC and 12 that were positive by real-time PCR and negative by BC. The negative real-time PCR results that were positive by BC failed to show frequent bloodstream pathogens such as CoNS, enterococci, streptococci and *K. pneumoniae*. Varani et al. have reported positive BC results for CoNS and *Streptococcus mitis*, which were not detected by real-time PCR [14]. Louie et al. have detected positive BC for *Enter. Faecalis*, whereas real-time PCR failed to identify this microorganism [15]. This difference allowed us to establish a limit of detection (LoD) to distinguish between infection and contamination. The low sensitivity of real-time PCR for CoNS, *Enterococcus* spp. and *Str. pneumoniae* was possibly associated with the C*t* and LoD. C*t* specifically sets analytical cutoff values to distinguish contamination from infection in bone marrow transplant patients. Lehmann et al. have reported the importance of determining cutoff values to discriminate between significant bacterial loads in clinical samples and low amounts of bacterial DNA [16]. In addition, the presence of a high bacterial DNA concentration might inhibit molecular detection [17].

In samples in which bacteria were only detected with real-time PCR, the microorganisms detected were one *A. baumannii*, two *K. pneumoniae*, one *Enterococcus* spp. and three CoNS (Table 1). Varani et al. have detected Gram-negative bacteria more than BC and *Enterococcus* spp. did not grow in culture [14]. Louie et al. have observed 17 cases in which PCR identified an organism that was not found by BC [15]. The previous use of antibiotics that is common in our patient population, including antibiotic prophylaxis, could contribute for these cases of bacteria only detected with real time PCR. Our cases that were positive for *K. pneumoniae* and *A. baumannii* showed genuine bacterial recovery because 19 days after PCR, *K. pneumoniae* was detected in another culture that was not available for real-time PCR. Others have suggested that the detection of circulating bacterial DNA and the presence of nonviable bacterial DNA detected by real-time PCR could be considered as contaminants [16,18].

The real-time PCR detected *Candida* DNA in three samples from two patients with probable or possible BSI, whereas BC showed negative results. Differences among BC systems for detection of *Candida* spp. do not explain the disagreement with the real-time PCR [19,20]. Mancini et al. have detected *Aspergillus* spp. in blood samples by LightCycler SeptiFast (Roche Diagnostics, GmbH, Mannheim, Germany) [13]. The identification by real-time PCR was consistent with the presence of nonviable fungal components or of very low viable fungal loads [21,22].

The aim of our study was to detect 10 resistance genes. We found samples with *bla*SHV, *bla*CTX-M, *van*A, and *mec*A genes by real-time PCR. Identification of these genes, together with clinical context, could be an important tool to help with the management of the appropriate therapy. One of the limitations of commercial kits for identifying bacteria and fungi in blood using PCR is failure to provide antimicrobial susceptibility patterns and resistance genes [14]. In our study, real-time PCR did not detect all the resistance genes; therefore, real-time PCR does not replace conventional bacteriology for antimicrobial agents.

The nonexistence of a gold standard regular diagnostic procedure is a major limitation for the evaluation of new molecular techniques. The positive BC results due to contamination represent a limitation for the interpretation of positive or negative real-time PCR results [23]. Karahan et al. have reported false-positive BC results that were suggestive of the presence of microbial DNA [24]. Accordingly, a negative result by real-time PCR cannot exclude the presence of BSI in neutropenic patients, and Peters et al. and Nakamura et al. have recommended that interpretation of real-time PCR results should conform to the clinical context [23,25].

## Conclusion

BC remains the gold standard for microbial diagnostics. However, real-time PCR could also be a valuable tool. Every effort should be made to improve the yield of this new diagnostic modality, particularly in critically ill patients. The results obtained should be interpreted together with clinical and other laboratory data. A large controlled study is in progress to evaluate further the clinical benefits of using real-time PCR in this patient setting.

## Competing interests

The authors declare that they have no competing interests.

## Authors’ contributions

LCM participated in the design and coordination of the study, data analysis, and drafted the manuscript. TTR participated in the design of the study and carried out the PCR. KCB participated in the clinical data acquisition and carried out the Bactec experiments. CP participated in the design of the study. MGQ participated in the design of the study and carried out the PCR. FC participated in the design of the study. JSRO participated in the design of the study. ACCP participated in the design and coordination of the study and helped draft the manuscript. All authors read and approved the final manuscript.

## Pre-publication history

The pre-publication history for this paper can be accessed here:

http://www.biomedcentral.com/1471-2334/13/166/prepub
